# Knowledge, Attitudes, and Practices of Parents on Early Childhood Caries in Qatar—A Questionnaire Study

**DOI:** 10.1055/s-0041-1739446

**Published:** 2021-12-22

**Authors:** Aisha Saleh Al-Jaber, Hadeel Mohammad Al-Qatami, Feras Hasan Abed Al Jawad

**Affiliations:** 1 Pediatric Dentistry Section, Hamad Dental Center, Hamad Medical Corporation, Doha, Qatar; 2 Orthodontics Section, Hamad Dental Center, Hamad Medical Corporation, Doha, Qatar

**Keywords:** early childhood caries, knowledge, attitudes, practices, questionnaire, socioeconomic factor

## Abstract

**Objectives** The aims of the present study were to evaluate the level of knowledge, attitudes, and practices (KAP) toward early childhood caries (ECC) in a group of Qatari parents and to assess the association of sociodemographic factors on their KAP.

**Materials and Methods** A cross-sectional study which was based on a piloted self-administered questionnaire was conveniently distributed to parents who attended the Pediatric Dentistry Section, Hamad Dental Center (HDC), Doha, Qatar. The questionnaire comprised four parts which asked about sociodemographic characteristics, knowledge, attitudes, and practices. A score for each domain was given based on the percentage of correct answers.

**Statistical Analysis** Descriptive and analytical statistics were employed. For descriptive statistics, frequency of distribution in relation to sociodemographic characteristics and responses to items of the questionnaire were presented. For analytical statistics, associations between independent variables and KAP were employed using Chi-squared tests.

**Results** The overall mean scores of KAP were 60.8%, 65.6% and 72.7%, respectively. Females had significantly higher percentages of correct answers than males ( *p* = 0.001). Only 20% of females had poor knowledge, while it was 40% in males. Parents with university or higher degrees had significantly higher percentage of good attitudes than parents with preparatory or less education ( *p* = 0.05). Areas that necessitated improvement by parents included the following: the amount of toothpaste needed for brushing, signs of tooth demineralization, bacteria that causes tooth decay can be transmitted from mother to her child, and tooth decay can be transmitted by sharing utensils (i.e., spoons, forks).

**Conclusions** The overall KAP of parents toward ECC was relatively fair. However, certain socioeconomic factors (SEF) seemed to influence each domain, and areas of improvement are needed. Areas of improvement are needed in each domain. Mothers were significantly more knowledgeable than fathers regarding oral health issues of their children. Highly educated parents demonstrated better attitudes than the less educated. Continuous educational programs coordinated by health regulatory bodies should be introduced to improve parents' KAP regarding ECC risk factors and prevention.

## Introduction

The oral cavity is an important part of the body, as it is one of the major focal points for the interaction of the body with the external environment. As a result, oral health is an integral component of general health and well-being. 1 Dental caries is one of the most widespread noncommunicable diseases in children and remains a major public health problem worldwide. 2_._ According to the American Academy of Pediatric Dentistry (AAPD), early childhood caries (ECC) is defined as “the presence of one or more decayed (non cavitated or cavitated lesions), missing (due to caries), or filled tooth surfaces in any primary tooth in a child 71 months of age or younger.” 3

ECC progresses rapidly, and if it remains untreated, it might cause chronic pain, altered speech, altered appetite, difficulty in chewing, weight loss, malnutrition and difficulty in sleeping. 4 Such problems can lead to negative impacts on growth and development. 4 5 Further progression without treatment can lead to tooth loss, which will compromise the future of permanent dentition eruption and occlusion. 4 6 Other negative consequences include increased risk of hospitalizations and emergency visits, increased treatment cost, and loss of school days. 4 Furthermore, it is well reported that the quality of life of preschool children is negatively affected due to ECC. 7

The etiology of ECC is a result of the interaction of bacteria, mainly Streptococcus mutans (SM), with the fermentable carbohydrate on a susceptible tooth structure over a specific duration. 8 However, recent efforts have focused on social determinants that influence the development and progression of ECC and not only biological factors. 9 Social determinants can shape the child's behaviors and attitudes. 9 Adopting positive behaviors that will ensure good oral hygiene and less consumption of cariogenic diet are one of the key factors in the prevention of ECC. 10 It is well-known that early habits and behaviors adopted at younger age can persist at an older age and these acquired behaviors are influenced by parents' beliefs and attitudes. 11 Fisher-Owens et al introduced a conceptual model of the influences on children's oral health. 12 Among the most important levels that were proposed was the family influence as part of the whole community. 11 Hence, parents' knowledge and beliefs are very important in shaping their children's future behaviors. 11 13 14 One of these is promoting positive behaviors that would lead to maintaining an intact oral health. 13

The fact that parents are solely responsible for their child's oral health care has led several studies to elucidate the knowledge, attitudes, and practices (KAP) of parents in different populations as one of the key social determinants of oral health. 15 16 17 18 19 Some studies reported poor knowledge of parents. 16 18 20 21 Others found good knowledge and attitudes but suboptimal practices. 15 19 22 23 However, most studies concluded that educational programs and initiatives are needed to increase the level of parents' awareness regarding oral health issues. 18 19 20 21 22 Moreover, distal factors such as socioeconomic factors (SEF) seemed to influence the level of KAP, as several studies reported that parents of higher SEF demonstrated better KAP level toward children's oral health. 24 Hence, the traditional biological and dietary approaches in investigating the etiology of ECC in isolation of other factors such as family factors and SEF seem to be not adequate. 21

In Qatar, the prevalence of ECC is reported to be 89.2% in preschool children aged 4 to 5 years old. 25 This alarming figure has led local health regulatory bodies to call for immediate actions that would warrant improvement in oral hygiene behaviors among children, promote preventive programs, and increase the level of awareness in the public. Since parents play a pivotal role in shaping their children's oral hygiene behaviors and habits, investigating the level of their KAP toward ECC would provide an insight about whether oral health promotion, education, and motivation are needed to reinforce their KAP and encourage healthy oral preventive measures for their children. 13 This, in turn, will support the efforts targeted in lowering the prevalence of ECC. Therefore, the aims of the present study were to evaluate the level of KAP toward ECC in a group of Qatari parents and assess the role of sociodemographic factors on their KAP.

## Materials and Methods

The study was presented in accordance with the Strengthening the Reporting of Observational Studies in Epidemiology statement (STROBE). 26

### Study Design

Cross-sectional study. Ethical approval was obtained from the Medical Research Center (MRC) at Hamad Medical Corporation (HMC), Doha, Qatar (MRC-01–19–161).

### Participants and Setting

The study population included parents who attended, between March to December 2019, the Pediatric Dentistry Section, Hamad Dental Center (HDC), HMC, Doha, Qatar. Parents were consecutively recruited (convenient sampling) and met the following inclusion criteria: primary caretakers with healthy children who were getting treated for dental caries, and child's age should be 71 months of age or below. 3 Parents who were unable to respond were excluded. Participation was voluntary, and each parent was given an information sheet describing the objectives of the study. Written informed consent was obtained if the parent agreed to participate.

### Outcomes

The primary outcomes were levels of parents' KAP. Sociodemographic variables were considered predictors (independent variables), which included age, gender, education level, employment status and income.

### Questionnaire

A structured self-administered questionnaire was used. Items of the questionnaire were adopted from previous research done on this topic, with modifications related to the number of items and response options. 18 27 To ensure that items of the questionnaire are equivalent to the original items in English language and that it had an acceptable content validity, the following steps were implemented:

Forward translation: That was done by asking two experienced health professionals in research and questionnaires development, who were not involved in the study and were fluent in English and Arabic languages to translate the items to Arabic language by taking into account the conceptual equivalent of a word or phrase, making it suitable, simple, and concise. Any discrepancies related to the concept/expression translations were resolved by arranging a meeting.

Backward translation: Items were then back translated to English language, using the same approach as forward translation.

Pretesting: The questionnaire was tested in 25 parents (10 males, 15 females) to assess feasibility and clarity. No issues were reported with respect to wording, concepts, number of items, and time needed to complete the questionnaire. In addition, 10 out of the 25 were asked to refill the questionnaire after 2 weeks to assess reliability, which was excellent, according to the intraclass correlation coefficient (ICC = 0.89) test.

Construct validity could not be tested in relation to KAP, as they were not psychosocial constructs in which psychometric properties need to be tested. The present study evaluated outcomes in relation to a dental problem that has specific guidelines from the literature on how to prevent it. Furthermore, all studies that evaluated parents' KAP did not report on the construct validity, instead they used the same items but with differences in wording, number, or options. Content validity was assessed by asking experts in questionnaire.

The final version of the questionnaire consists of the following four domains:

The first domain assessed sociodemographic data, which included age, gender, educational level, number of children, and employment income. Age was categorized into 20 to 30, > 30 to 40, and > 40 years; education was categorized into preparatory and less, high school, university, higher education, the total number of children, the number of children who were 6 years or below, and the income which was categorized to < 5000 QR ∼ 1150 €, 5000 to 9999 QR ∼ 1151 to 2300 €, 10000 to 19999 QR ∼ 2301 to 4600 €, 20000 to 29999 QR ∼ 4601 to 6900 €, 30000 to 39999 QR ∼ 6901 to 9200€, and ≥ 40000 QR ∼ ≥ 9201€.

The second domain assessed the knowledge of parents using 15 items which covered general knowledge about the amount of toothpaste applied according to the age, effect of mother's diet on her child's oral health, frequency of canned juices and sugary intake, the time of the first dental visit, the importance of fluoride, the appearance of white lines/spots on teeth surfaces, and preventive measures. Options for each item were “yes,” “no,” and “I don't know,” where “yes” corresponds to a positive knowledge.

The third domain assessed attitudes using 7 items which covered parents' responsibility toward their children oral health, the effect of prolonged and frequent breast feeding, providing fresh juices frequently, dental checkup once the first tooth erupts, and parent/caregiver help for their children oral hygiene. A five-point Likert scale options were used ranging from “strongly agree” to “strongly disagree” where “strongly agree” or “agree” reflect positive attitude.

The fourth domain assessed practices using five items which covered practices related to balanced diet and its relation to their children oral health, providing breastfeeding/bottle feeding during bedtime, transmission of SM by sharing utensils, need of efforts to improve their knowledge in oral health, and the necessity in dental cleaning after each meal. Similar to attitudes domain, the same five-point Likert scale options were used.

### Bias

To avoid the influence of investigators, parents filled out the questionnaire in a special quiet room alone on site. Data collection was done by one of the investigators (A.A.), who adopted a standardized protocol with respect to approaching parents, taking consent, and administering the questionnaire. In addition, data entry and analysis were done by independent people who were not involved in the study.

### Sample Size Calculation

The level of parental KAP ranged from 45 to 65% in previous studies; 19 21 27 thus, an estimate of 50% was used with 6% margin of error, 95% confidence interval (CI) and 80% power. Therefore, the sample size for this study was 270 participants. However, to compensate for possible incomplete data, the sample size was inflated to 300 participants.

The following statistical formula was used:

n = [Z ^2^_1-α/2_ P(1-P) / d ^2^ ]

Where n = sample size,

Z = Z statistic for a level of confidence

(for the level of confidence of 95%, which is conventional, Z value is 1.96)

P = expected prevalence or proportion

d = precision (in proportion of one; if 5%, d = 0.05).

### Statistical Analysis

Descriptive and analytical statistics were employed. For descriptive statistics, frequency of distribution in relation to sociodemographic data and responses to items of the questionnaire were presented. For analytical statistics, associations between the independent variables and KAP were employed using Chi-square test. Scoring of KAP was based on the percentage of the correct answers. For knowledge, the response “yes” was considered a correct answer, whereas responses “no” or “I don't know” were considered incorrect answers. For attitudes and practices, “strongly agree” or “agree” were considered correct answers and the rest were considered incorrect answers. The percentage of correct answers for each domain was calculated by dividing the number of correct answers to the maximum possible number of correct answers multiplied by 100. A percentage of 49 or below was considered poor, 50 to 69% fair, and ≥ 70% was considered good.

*p* value was set on 0.05 and SPSS software (version 22) was used for data analysis. 28

## Results

### Sociodemographic Characteristics

Out of the 300 who were invited to participate, a total of 277 agreed to participate and filled in the questionnaire; thus, the response rate was 92.3%. The mean age of the sample was 37.3 (±1.4) and the majority were females 203 (73.3%). Most were married 269 (94%) and more than half had university or higher education degrees (133 [48%] and 20 [7.2%], respectively). Almost quarter of the parents had 3 children 67 (24%) and third of them 69 (34.7%) had 2 children who are 6 years old or below ( Table 1 ).

**Table 1:** Sociodemographic characteristics of the participants

	Number	%
Gender		
Female	203	73.3
Male	74	26.7
Marital status		
Married	260	93.9
Divorced	8	2.9
Missing	9	3.2
Educational level		
Preparatory and less	28	10.1
High school	91	32.9
University	133	48.0
Higher education	20	7.2
Missing	5	1.8
Average salary		
< 5000 QR	6	2.2
5000–9999 QR	31	11.2
10000–19999 QR	60	21.7
20000–29999 QR	43	15.5
30000–39999 QR	38	13.7
≥ 40000 QR	42	15.2
Missing	57	20.6
Number of children		
1	12	4.3
2	39	14.1
3	67	24.2
4	43	15.5
5	38	13.7
6	25	9
7	10	3.6
8	7	2.5
9	4	1.4
10	2	0.7
Missing	30	10.8
Number of children 6 years of age and below		
1	78	28.2
2	96	34.7
3	48	17.3
4	6	2.2
6	2	0.7
Missing	47	17

The overall mean scores of KAP were 60.8%, 65.6% and 72.7%, respectively ( Tables 2 , 3 , 4 ).

**Table 2:** Frequencies of knowledge items

No.	Questions	Correct *n* (%)	Incorrect *n* (%)
1 and 2	Quantity of toothpaste in children < 3 years	181 (65.3%)	96 (34.7%)
3 and 4	Quantity of toothpaste in children > 3 years	237 (85.6%)	40 (14.4%)
5	Mothers' diet during pregnancy affects development of baby's teeth	173 (62.5%)	104 (37.5%)
6	Canned juice can be given frequently to your child	16 (5.8%)	261 (94.2%)
7	Dental checkup at first year of your child life is important even if child does not have tooth decay or tooth pain	212 (76.5%)	64 (23.1%)
8	Feeding your child with baby bottle at nighttime has an influence on child teeth	211 (76.2%)	65(23.5%)
9	Fluoridated toothpaste helps in preventing your child tooth decay	185(66.8%)	91 (32.9%)
10	Controlling sugary intake frequency during the day can affect tooth decay	258 (93.1%)	18 (6.5%)
11	Appearance of white lines or white spots on the surfaces of the teeth are the first signs of tooth decay	134 (48.4%)	143 (51.6%)
12	Germs that cause tooth decay can be transmitted from mother to her child by kissing on his lips/chewing food by mother before giving it to her child	102 (36.8%)	175 (63.2%)
13	Decay in baby teeth can harm the future of new adult teeth	127 (45.8%)	148 (53.4%)
14	the care of the oral health and oral hygiene is important for the health of the permanent teeth	247 (89.2%)	30 (10.8%)
15	Tooth decay in children is inherited	48 (17.3%)	229 (82.7%)
Overall mean of knowledge	60.8%		

Note: Option: “yes” considered as correct item, while “no” or “don't know” considered as incorrect item.

**Table 3:** Frequencies of attitude items

No.	Questions	Correct *n* (%)	Incorrect *n* (%)
1	Maintaining the child oral health is the parent's responsibility	267 (96.4%)	10 (3.6%)
2	Prolonged and frequent breast-feeding harm your child's teeth	69 (24.9%)	208 (75.1%)
3	Providing fresh juices frequently during the day can harm your child's teeth	110 (39.7%)	167 (60.3%)
4	Baby teeth should be cleaned as soon as it erupts	213 (76.9%)	64 (23.1%)
5	Taking the child for dental check-up as soon as his/her teeth erupt	171 (61.7%)	106 (38.3%)
6	Brushing the children's teeth at the age of 6 years and below should be done under the help of parents/caregiver	261 (94.2%)	16 (5.8%)
Overall mean of attitude	65.6%		

**Table 4:** Frequencies of practice items

No.	Questions	Correct *n* (%)	Incorrect *n* (%)
1	Balanced diet is necessary for your child oral health	268 (96.8%)	9 (3.2%)
2	Providing breast feeding /bottle feeding during bed-time could harm your child teeth	166 (59.9%)	111 (40.1%)
3	Tooth decay can be transmitted by sharing utensils (i.e., spoons, forks)	61 (22%)	216 (78%)
4	Parents should make an effort to improve their knowledge in oral health	267 (96.4%)	10 (3.6%)
5	Cleaning your child's teeth after each meal is necessary	245 (88.4%)	32 (11.6%)
Overall mean of practice	72.7%		

Note: Options of attitude and practice”: strongly agree” or “agree” considered correct, while “strongly disagree,” “disagree” and “don't know” considered incorrect.

### Knowledge

Gender was the only variable that had a significant association with knowledge ( *p* = 0.001). Females had significantly higher percentages of correct answers than males ( Table 5 ). Almost 34% of females had good knowledge, while it was only 8.2% for males. Only 20% of females had poor knowledge, while it was 40% in males. Almost half of females and males had fair knowledge ( Table 5 ).

**Table 5:** The association between knowledge and SEF in early childhood caries

Variables		Knowledge				
		Good *n* (%)	Fair *n* (%)	Poor *n* (%)	Total *n*	*p* -Value (Chi-square)
Gender	Female	56 (33.9%)	76(46.1%)	33(20.0%)	165	0.000
	Male	5 (8.2%)	32(52.5%)	24(39.3%)	61	
	Missing	51 (18.4%)				
Marital status	Married	59 (27.4%)	104(48.4%)	52(24.2%)	215	0.797
	Divorced	1 (16.7%)	3(50%)	2(33.3%)	6	
	Missing	56 (20.2%)				
Nationality	Qatari	48 (27.1%)	87(49.2%)	42(23.7%)	177	0.577
	Non-Qatari	10 (26.3%)	16(42.1%)	12(31.6%)	38	
	Missing	62 (22.4%)				
Age	19–29 years	12 (30%)	19(47.5%)	9(22.5%)	40	0.811
	30–39 years	29 (24.4%)	60(50.4%)	30(25.2%)	119	
	≥ 40 years	18 (29%)	26(41.9%)	18(29%)	62	
	Missing	56 (20.2%)				
Number of children	1	4 (40.0%)	6 (60.0%)	0 (0.0%)	10	0.240
	2	5(16.7%)	12 (40.0%)	13 (43.3%)	30	
	3	14 (25.0%)	27 (48.2%)	15 (26.8%)	56	
	4	9 (27.3%)	18 (54.5%)	6 (18.2%)	33	
	5	8 (22.9%)	19 (54.3%)	8(22.9%)	35	
	6	5 (23.8%)	10 (47.6%)	6 (28.6%)	21	
	7	2(22.2%)	6 (66.7%)	1(11.1%)	9	
	8	3 (42.9%)	2 (28.6%)	2(28.6%)	7	
	9	0 (0.0%)	0 (0.0%)	1 (100.0%)	1	
	10	2 (100.0%)	0 (0.0%)	0 (0.0%)	2	
	Missing	73 (26.4%)				
Children ≤ 6yrs	1	19(29.2%)	34(52.3%)	12 (18.5%)	65	0.414
	2	15(18.8%)	36 (45.0%)	29 (36.3%)	80	
	3	11 (30.6%)	16 (44.4%)	9 (25.0%)	36	
	4	2 (33.3%)	3 (50.0%)	1 (16.7%)	6	
	6	0 (0.0%)	1 (50.0%)	1 (50.0%)	2	
	Missing	88 (31.8%)				
Educational level	Preparatory and less	5 (22.7%)	9 (40.9%)	8(36.4%)	22	0.274
	High school	16 (22.2%)	37 (51.4%)	19(26.4%)	72	
	University	33 (28.7%)	58 (50.4%)	24 (20.9%)	115	
	Higher education	5 (35.7%)	3 (21.4%)	6 (42.9%)	14	
	Missing	54 (19.5%)				
Income	< 5000 QR	3 (50.0%)	1 (16.7%)	2 (33.3%)	6	0.136
	5000–9999 QR	3 (13.6%)	13 (59.1%)	6(27.3%)	22	
	10000–19999 QR	20 (40.8%)	19 (38.8%)	10 (20.4%)	49	
	20000–29999 QR	6 (18.2%)	21(63.6%)	6 (18.2%)	33	
	30000- 39999 QR	9 (26.5%)	14 (41.2%)	11 (32.4%)	34	
	≥ 40000	8 (22.2%)	16 (44.4%)	12 (33.3%)	36	
	Missing	97 (35%)				

Abbreviation: SEF, socioeconomic factor.

Items with the most prevalent incorrect answers for knowledge included the following: the amount of toothpaste needed for brushing (67%), the appearance of white lines or white spots on the surfaces of the teeth are the first signs of tooth decay (52%), and germs that cause tooth decay can be transmitted from mother to her child (54%).

### Attitudes

Educational level was the only variable that was significantly associated with attitudes ( *p* = 0.05). Parents with university or higher degrees had significantly higher percentage of good attitudes (37% and 30%, respectively) than parents with preparatory or less education (18%, Table 6 ). They also had lower percentages of poor attitudes (10.5% and 10%, respectively) when compared with parents with preparatory or less education (28%). Most parents correctly answered the questions pertaining to maintaining child's oral health is the parent's responsibility and brushing children's teeth at the age of 6 years and below should be done under the supervision of parents/caregiver (96.4 and 94.6, respectively). Items with the most incorrect answers included the following: prolonged and frequent breastfeeding or providing fresh juices frequently during the day can harm your child's teeth (75% and 60%, respectively).

**Table 6:** The association between attitude and SEF in early childhood caries

Variables		Attitude				
		Good *n* (%)	Fair *n* (%)	Poor *n* (%)	Total *n*	*p* -Value (Chi-square)
Gender	Female	63(31.3%)	116	22	201	0.483
	Male	23 (31.1%)	(57.7%)	(10.9%)	74	
	Missing	2 (0.7%)	39 (52.7%)	12 (16.2%)		
Marital status	Married	78 (30%)	150	32	260	0.894
	Divorced	3 (37.5%)	(57.7%)	(12.3%)	8	
	Missing	9 (3.2%)	4 (50.0%)	1 (12.5%)		
Nationality	Qatari	59 (27.7%)	122 (57.3%)	32 (15.0%)	213	0.030
	Non-Qatari	19 (38.8%)	29 (59.2%)	1 (2.0%)	49	
	Missing	15 (5.4%)				
Age	19–29 years	10 (18.9%)	38 (71.7%)	5 (9.4%)	53	0.139
	30–39 years	50 (34.5%)	75 (51.7%)	20 (13.8%)	145	
	≥ 40 years	25 (35.2%)	38 (53.5%)	8 (11.3%)	71	
	Missing	8 (2.9%)				
Number of children	1	3 (8.3%)	8 (66.7%)	1 (8.3%)	12	0.983
	2	10(25.6%)	23(59.0%)	6 (15.4%)	39	
	3	21 (31.3%)	40 (59.7%)	6 (9.0%)	67	
	4	12(27.9%)	27(62.8%)	4 (9.3%)	43	
	5	12 (31.6%)	21 (55.3%)	5 (13.2%)	38	
	6	9 (36.0%)	12 (48.0%)	4 (16.0%)	25	
	7	4 (40.0%)	5 (50.0%)	1 (10.0%)	10	
	8	2 (28.6%)	3 (42.9%)	2 (28.6)	7	
	9	1 (25.0%)	2 (50.0%)	1 (25.0%)	4	
	10	0 (0.0%)	2 (100.0%)	0 (0.0%)	2	
	Missing	47 (17.0%)				
Children ≤ 6yrs	1	29 (37.2%)	39 (50.0%)	10 (12.8%)	78	0.340
	2	23 (24.0%)	59 (61.5%)	14 (14.6%)	96	
	3	19 (39.6%)	25 (52.1%)	4 (8.3%)	48	
	4	1 (16.7%)	4 (66.7%)	1 (16.7%)	6	
	6	0 (0.0%)	1 (50.0%)	1 (50.0%)	2	
	Missing	30 (10.8%)				
Educational level	Preparatory and less	5 (17.9%)	15 (53.6%)	8 (28.6%)	28	0.05
	High school	25 (27.5%)	56 (61.5%)	10 (11.0%)	91	
	University	50 (37.6%)	69 (51.9%)	14 (10.5%)	133	
	Higher education	6 (30.0%)	12 (60.0%)	2 (10.0%)	20	
	Missing	5 (1.8%)				
Income	< 5000 QR	2 (33.3%)	3 (50.0%)	1 (16.7%)	6	0.080
	5000–9999 QR	14 (45.2%)	12 (38.7%)	5 (16.1%)	31	
	10000–19999 QR	18 (30.0%)	37 (61.7%)	5 (8.3%)	60	
	20000–29999 QR	5 (11.6%)	31 (72.1%)	7 (16.3%)	43	
	30000- 39999 QR	10 (26.3%)	25 (65.8%)	3 (7.9%)	38	
	≥ 40000	17 (40.5%)	19 (45.2%)	6 (14.3%)	42	
	Missing	57 (20.6%)				

Abbreviation: SEF, socioeconomic factor.

### Practices

There were no significant associations between any of the sociodemographic variables and practice scores ( Table 7 ). The majority of parents provided correct answers for items relating to balanced diet is necessary for your child oral health and parents should make an effort to improve their knowledge on oral health (97% and 94.6%, respectively). However, with respect to the item relating to tooth decay can be transmitted by sharing utensils (i.e., spoons, forks), most of the parents provided incorrect answer (78%).

**Table 7:** The association between practice and SEF in early childhood caries

Variables		Practice				
		Good *n* (%)	Fair *n* (%)	Poor *n* (%)	Total *n*	*p* -Value (Chi-square)
Gender	Female	121 (60.2%)	68 (33.8%)	12(6.0%)	201	0.956
	Male	45 (60.8%)	24 (32.4%)	5 (6.8%)	74	
	Missing	2 (0.7%)				
Marital status	Married	156 (60.0%)	87 (33.5%)	17 (6.5%)	260	0.752
	Divorced	5 (62.5%)	3 (37.5%)	0 (0.0%)	8	
	Missing	9 (3.2%)				
Nationality	Qatari	128 (60.1%)	70 (32.9%)	15 (7.0%)	213	0.605
	Non-Qatari	28 (57.1%)	19 (38.8%)	2 (4.1%)	49	
	Missing	15 (5.4%)				
Age	19–29 years	32 (60.4%)	16 (30.2%)	5 (9.4%)	53	0.786
	30–39 years	89 (61.4%)	49 (33.8%)	7 (4.8%)	145	
	≥ 40 years	41 (57.7%)	25 (35.2%)	5 (7.0%)	71	
	Missing	8 (2.9%)				
Number of children	1	7 (58.3%)	5 (41.7%)	0 (0.0%)	12	0.732
	2	26 (66.7%)	12 (30.8%)	1 (2.6%)	39	
	3	45 (67.2%)	19 (28.4%)	3 (4.5%)	67	
	4	24 (55.8%)	15 (34.9%)	4 (9.3%)	43	
	5	24 (63.2%)	12 (31.6%)	2 (5.3%)	38	
	6	12 (48.0%)	10 (40.0%)	3 (12.0%)	25	
	7	5 (50.0%)	3 (30.0%)	2 (20.0%)	10	
	8	6 (85.7%)	1 (14.3%)	0 (0.0%)	7	
	9	2 (50.0%)	2 (50.0%)	0 (0.0%)	4	
	10	2 (100%)	0 (0.0%)	0 (0.0%)	2	
	Missing	30 (10.8%)				
Children ≤ 6 years	1	54 (69.2%)	19 (24.4%)	5 (6.4%)	78	0.375
	2	56 (58.3%)	35 (36.5%)	5 (5.2%)	96	
	3	33 (68.8%)	12 (25.0%)	3 (6.3%)	48	
	4	4 (66.7%)	2 (33.3%)	0 (0.0%)	6	
	6	0 (0.0%)	2 (100.0%)	0 (0.0%)	2	
	Missing	47 (17.0%)				
Educational level	Preparatory and less	13 (46.4%)	11	4	28	0.343
	High school	55 (60.4%)	(39.3%)	(14.3%)	91	
	University	82 (61.7%)	29 (31.9%)	7(7.7%)	133	
	Higher education	14 (70.0%)	45 (33.8%)	6 (4.5%)	20	
	Missing	5 (1.8%)	6 (30.0%)	0 (0.0%)		
Income	< 5000 QR	4 (66.7%)	1 (16.7%)	1 (16.7%)	6	0.399
	5000–9999 QR	17 (54.8%)	9 (29.0%)	5 (16.1%)	31	
	10000–19999 QR	33 (55.0%)	24 (40.0%)	3 (5.0%)	60	
	20000–29999 QR	28 (65.1%)	12 (27.9%)	3 (7.0%)	43	
	30000- 39999 QR	22 (57.9%)	15 (39.5%)	1 (2.6%)	38	
	≥ 40000	29 (69.0%)	11 (26.2%)	2 (4.8%)	42	
	Missing	57 (20.6%)				

Abbreviations: *n* , number; SEF, socioeconomic factor.

## Discussion

In 2018, a meeting was held by oral health experts “A WHO Global Consultation on ECC” to provide an overview of ECC prevention strategies and emphasize the importance of actions needed to control the spread of this disease. 29 Among their recommendations was to focus on families/caregivers taking into consideration their sociobehavioral aspects as well as personal factors as part of health policies that aim for oral diseases prevention, oral health promotion, and allocating resources. 29 Parents and caregivers play a major role in reinforcing positive oral health behaviors such as eating healthy foods with less sugar intake, adopting measures to improve oral hygiene and making regular dental visits. 13 Therefore, to achieve these goals, assessing the KAP of parents toward ECC would be beneficial and would also increase our understanding toward actions and interventional approaches that are needed to prevent this public health problem.

In this study, the knowledge of parents was considered to be fair (61%), which is in line with previous studies whose populations share similar cultural characteristics. 2 23 30 However, reports from other populations revealed better levels of knowledge. 19 This might be attributed to factors related to literacy or other environmental factors that could influence knowledge levels between different populations. For example, Vann et al found in a group of female caregivers that lower caregiver literacy was associated with deleterious oral health behaviors such as nighttime bottle-feeding use and no daily brushing of teeth. 31

According to the findings of this study, mothers were significantly more knowledgeable than fathers. This finding coincides with results found in a group of Kuwaiti parents. 16 This outcome is expected, as mothers are generally more involved in the daily care of their children, including oral hygiene care. 16 Moreover, mothers are more involved in their children's health care appointments which, in turn, make them more exposed to health care professionals. As a result, this will allow them to learn more about their children's dental health. Nevertheless, this study emphasizes that the role of fathers should be improved, and every effort should be made to increase their knowledge levels with respect to oral health issues. That is, by increasing the level of their awareness by adopting several approaches and utilizing the media or providing educational programs and information leaflets for parents at schools.

This study identified strengths in the knowledge of parents such as questions concerning dental checkups at first year, the effect of drinking canned juices frequently, the effect of controlling the frequency of sugar intake during the day, the importance of the primary teeth for the health of permanent teeth, and whether tooth decay is inherited. Most of the parents answered the questions correctly (75.5%, 94.2%, 93%, 89% and 83%, respectively). These finding were comparable to other populations in the same region. 16 20 23 27 30 Contrary to our findings, a previous study conducted in a group of Qatari parents found that 64% of mothers did not know the timing of the first dental visit. 2 Thus, it would appear based on the present study that good improvement in parents' knowledge has occurred. Another study in a group of Saudi parents found that 53% did not consider taking their children to the dentist when they are 1 year old. 32

Areas of knowledge weaknesses were also identified in the present study. The majority of parents did not answer correctly the question concerning the quantity of toothpaste that should be given to the child for teeth brushing. This finding coincided with several studies in different populations. 33 34 According to recent evidence, the ideal amount of fluoridated toothpaste in children is one of the most important preventive measures in reducing ECC. 35 Creeth et al recommended using a pea-sized toothpaste in children from 3 to 6 years of age. 36 For children who are 2 years old or less, the AAPD recommends using of a “smear” of fluoride toothpaste (∼ 0.1 g of toothpaste or 0.1 mg of fluoride). 3 It would appear that parents need supporting educational programs about the importance of the amount of toothpaste to be used by their child and how much is enough. Another concerning finding of this study was that more than half of the parents were unaware that white lines or spots on their children's teeth were the first indicators of dental caries. It is well known that dental caries is a preventable disease, and if detected at an earlier stage, further progression can be prevented. 3 4 8 Thus, parents should be educated about the first clinical signs of tooth decay and instructed to seek professional dental consultation once early signs are found.

With respect to parents' attitudes, the overall score was also considered fair (65.6%), although some improvement is needed in certain areas. For example, only quarter of the parents agreed that prolonged and frequent breastfeeding can harm child's teeth, and this finding was in accordance with another studies. 16 19 In addition, only 40% of parents correctly answered the question relating to providing fresh juices frequently during the day can harm the child's teeth. Although juices with added sugar causes dental caries, 100% fresh juices can lead to erosion and demineralization of the teeth if consumed frequently. 37 Thus, parents should be educated about possible deleterious effects of naturally occurring sugars in fresh juices on tooth structure and encouraging their children to adopt healthy eating behaviors.

Educational level was the only socioeconomic variable that was significantly associated with parents' attitudes. That is, highly educated parents had higher levels of attitudes and compared with the less educated. This finding was in agreement with another sample of Saudi parents. 27 It is well reported that socioeconomic determinants, such as educational level of parents, are associated with caries experience in children. Hooley et al concluded that lower family income and parental education were associated with higher risk of dental caries in children aged 0 to 6 years. 38 Based on our findings, initiatives that aim to target parents with lower educational backgrounds should improve their attitudes toward oral hygiene care and ECC prevention.

Practices of parents toward oral health care were assessed, as it is a realistic reflection of both knowledge and attitudes. Although few studies reported that good knowledge and attitudes did not positively affect parents' practices, 19 the present study demonstrated good practices of parents toward their children's oral hygiene care (72%). The vast majority of parents gave correct answer to the question relating to balanced eating and healthy and less sugary diet is necessary to their children's oral health. Furthermore, most of the parents answered positively regarding making efforts to improve their knowledge on oral health issues. This was an encouraging finding and reflected the willingness of parents to improve their knowledge to pursue healthy practices related to their children's oral hygiene. However, and with respect to the question related to whether tooth decay can be transmitted by sharing utensils (i.e., spoons, forks), most of the parents gave incorrect answers (78%), which was in agreement to another study elsewhere. 20 26 It is well-known that dental caries is an infectious disease, and earlier studies demonstrated that infants acquire SM from their mothers by biting the food into small pieces before feeding, kissing, or sharing utensils, only after the eruption of primary teeth. 4 Therefore, mothers should be educated about the vertical and horizontal transmissions of SM and avoid sharing activities in which SM might be acquired.

Despite the strengths of the present study, such as the large sample size, the outcomes investigated and evaluating the role of sociodemographic on parents' KAP, the study had limitations. Adopting convenient sampling and recruiting parents from a single center might have biased the results and affected the generalizability of our findings. Moreover, the cross-sectional design of the present study may preclude the establishment of casual relationship between sociodemographic factors and KAP.

It would appear based on the findings of this study that the level of parents' KAP toward their children's oral health is acceptable when compared with a previous report in the same population. 2 However, certain areas of improvement in each domain are needed by promoting and planning policies that target parents with less educational backgrounds and improve fathers' knowledge. Moreover, educational programs coordinated by health regulatory bodies should be introduced to improve parents' knowledge regarding the risk factors of dental caries and early signs of tooth demineralization. 39 Mothers' attitudes regarding prolonged breastfeeding should be changed and informed about the risk of uncontrolled breastfeeding. Furthermore, the means of SM transmissions should be explained, in particular, following the eruption of the first primary tooth.

## Conclusions

The overall KAP of parents toward ECC was relatively fair. However, certain SEF seemed to influence each domain, and areas of improvement are needed. Mothers were significantly more knowledgeable than fathers regarding oral health issues of their children. Highly educated parents demonstrated better attitudes than the less educated. Modes of SM transmissions should be explained to parents. Finally, continuous educational programs coordinated by health regulatory bodies should be introduced to improve parents' KAP regarding ECC risk factors and prevention.

## References

[1] Baiju R M Peter E Varghese N O Sivaram R Oral health and quality of life: current concepts J Clin Diagn Res 2017 11 06 ZE21 ZE26 28764312 10.7860/JCDR/2017/25866.10110 PMC5535498

[2] Alkhtib A Morawala A Knowledge, attitudes, and practices of mothers of preschool children about oral health in Qatar: a cross-sectional survey Dent J (Basel) 2018 6 04 51 10.3390/dj6040051 PMC6313368 30275416

[3] American Academy on Pediatric Dentistry Policy on Early Childhood Caries (ECC): Classifications, Consequences, and Preventive StrategiesThe Reference Manual of Pediatric Dentistry 2020–2021:P. 79–81

[4] Colak H Dülgergil C T Dalli M Hamidi M M Early childhood caries update: a review of causes, diagnoses, and treatments J Nat Sci Biol Med 2013 4 01 29 38 23633832 10.4103/0976-9668.107257 PMC3633299

[5] Pakkhesal M Riyahi E Naghavi Alhosseini A Amdjadi P Behnampour N Impact of dental caries on oral health related quality of life among preschool children: perceptions of parents BMC Oral Health 2021 21 01 68 33588827 10.1186/s12903-021-01396-4 PMC7885600

[6] Luzzi V Fabbrizi M Coloni C Experience of dental caries and its effects on early dental occlusion: a descriptive study Ann Stomatol (Roma) 2011 2(1-2):13 18 22238717 PMC3254384

[7] Zaror C Matamala-Santander A Ferrer M Rivera-Mendoza F Espinoza-Espinoza G Martínez-Zapata M J Impact of early childhood caries on oral health-related quality of life: A systematic review and meta-analysis Int J Dent Hyg 2021(e-pub ahead of print) 10.1111/idh.12494 33825317

[8] Anil S Anand P S Early childhood caries: prevalence, risk factors, and prevention Front Pediatr 2017 5 157 28770188 10.3389/fped.2017.00157 PMC5514393

[9] Jain M Namdev R Bodh M Dutta S Singhal P Kumar A Social and behavioral determinants for early childhood caries among preschool children in India J Dent Res Dent Clin Dent Prospect 2015 9 02 115 120 10.15171/joddd.2014.023 PMC4517304 26236439

[10] Chu C H Chau A M Wong Z S Hui B S Lo E C Oral health status and behaviours of children in Myanmar - a pilot study in four villages in rural areas Oral Health Prev Dent 2012 10 04 365 371 23301237

[11] Castilho A R Mialhe F L Barbosa TdeS Puppin-Rontani R M Influence of family environment on children's oral health: a systematic review J Pediatr (Rio J) 2013 89 02 116 123 23642420 10.1016/j.jped.2013.03.014

[12] Fisher-Owens S A Gansky S A Platt L J Influences on children's oral health: a conceptual model Pediatrics 2007 120 03 e510 e520 17766495 10.1542/peds.2006-3084

[13] Duijster D de Jong-Lenters M Verrips E van Loveren C Establishing oral health promoting behaviours in children - parents' views on barriers, facilitators and professional support: a qualitative study BMC Oral Health 2015 15 157 26654364 10.1186/s12903-015-0145-0 PMC4676163

[14] Hiratsuka V Y Robinson J M Greenlee R Refaat A Oral health beliefs and oral hygiene behaviours among parents of urban Alaska Native children Int J Circumpolar Health 2019 78 01 1.586274E6 10.1080/22423982.2019.1586274 PMC6419661 30857502

[15] Mani S A Aziz A A John J Ismail N M Knowledge, attitude and practice of oral health promoting factors among caretakers of children attending day-care centers in Kubang Kerian, Malaysia: a preliminary study J Indian Soc Pedod Prev Dent 2010 28 02 78 83 20660972 10.4103/0970-4388.66741

[16] Ashkanani F Al-Sane M Knowledge, attitudes and practices of caregivers in relation to oral health of preschool children Med Princ Pract 2013 22 02 167 172 22986905 10.1159/000341764 PMC5586720

[17] Vanagas G Milasauskiene Z Grabauskas V Mickeviciene A Associations between parental skills and their attitudes toward importance to develop good oral hygiene skills in their children Medicina (Kaunas) 2009 45 09 718 723 19834309

[18] Dutta B Samir P V Knowledge, attitude, and practice of mothers towards infant oral healthcare Int J Clin Pediatr Dent 2018 11 05 435 439 30787559 10.5005/jp-journals-10005-1553 PMC6379533

[19] Suma Sogi H P Hugar S M Nalawade T M Sinha A Hugar S Mallikarjuna R M Knowledge, attitude, and practices of oral health care in prevention of early childhood caries among parents of children in Belagavi city: a questionnaire study J Family Med Prim Care 2016 5 02 286 290 27843829 10.4103/2249-4863.192332 PMC5084549

[20] BaniHani A Tahmassebi J Zawaideh F Maternal knowledge on early childhood caries and barriers to seek dental treatment in Jordan Eur Arch Paediatr Dent 2021 22 03 433 439 33210223 10.1007/s40368-020-00576-0 PMC8213663

[21] Dagon N Ratson T Peretz B Blumer S Maternal knowledge of oral health of children aged 1-4 years J Clin Pediatr Dent 2019 43 02 116 120 30730803 10.17796/1053-4625-43.2.8

[22] Wahengbam P P Kshetrimayum N Wahengbam B S Nandkeoliar T Lyngdoh D Assessment of oral health knowledge, attitude and self-care practice among adolescents - A state wide cross- sectional study in Manipur, north eastern India J Clin Diagn Res 2016 10 06 ZC65 ZC70 10.7860/JCDR/2016/20693.8002 PMC4963774 27504414

[23] Mahmoud N Kowash M Hussein I Hassan A Al Halabi M Oral health knowledge, attitude, and practices of Sharjah mothers of preschool children, United Arab Emirates J Int Soc Prev Community Dent 2017 7 06 308 314 29387613 10.4103/jispcd.JISPCD_310_17 PMC5774050

[24] Rai N K Tiwari T Parental factors influencing the development of early childhood caries in developing nations: a systematic review Front Public Health 2018 6 64 29616206 10.3389/fpubh.2018.00064 PMC5865069

[25] Alkhtib A Ghanim A Temple-Smith M Messer L B Pirotta M Morgan M Prevalence of early childhood caries and enamel defects in four and five-year old Qatari preschool children BMC Oral Health 2016 16 01 73 27539009 10.1186/s12903-016-0267-z PMC4989346

[26] STROBE Initiative von Elm E Altman D G Egger M Pocock S J Gøtzsche P C Vandenbroucke J P The Strengthening the Reporting of Observational Studies in Epidemiology (STROBE) statement: guidelines for reporting observational studies Bull World Health Organ 2007 85 11 867 872 18038077 10.2471/BLT.07.045120 PMC2636253

[27] Ravi Kumar G Ganji K K Patil S Alhadi A Alhadi M Parent's knowledge, attitude and practice on prevention of early childhood caries in Al Jouf province, Saudi Arabia Pesquisa Brasileira em Odontopediatria e Clínica Integrada 2018 18 01 e3837

[28] Daniel W W Biostatistics: A Foundation for Analysis in the Health Sciences 7th ed. Hoboken John Wiley & Sons, Inc 1999

[29] Phantumvanit P Makino Y Ogawa H WHO global consultation on public health intervention against early childhood caries Community Dent Oral Epidemiol 2018 46 03 280 287 29380407 10.1111/cdoe.12362

[30] Alshammary F Aljohani F A Alkhuwayr F S Siddiqui A A Measurement of parents' knowledge toward oral health of their children: an observational study from Hail, Saudi Arabia J Contemp Dent Pract 2019 20 07 801 805 31597799

[31] Vann W F Jr Lee J Y Baker D Divaris K Oral health literacy among female caregivers: impact on oral health outcomes in early childhood J Dent Res 2010 89 12 1395 1400 20924067 10.1177/0022034510379601 PMC3123718

[32] Togoo R A Bijle M N Al-Shahrani I Al-Absi W S Al-Shahrani F S Al-Shahrani A S Awareness among young parents about preventive aspects of early childhood caries in Abha City, Kingdom of Saudi Arabia World J Dent 2016 7 01 10 13

[33] Bennadi D Kshetrimayum N Sibyl S Reddy C V Toothpaste utilization profiles among preschool children J Clin Diagn Res 2014 8 03 212 215 24783140 10.7860/JCDR/2014/7309.4165 PMC4003646

[34] Naidu R S Nunn J H Oral health knowledge, attitudes and behaviour of parents and caregivers of preschool children: implications for oral health promotion Oral Health Prev Dent 2020 18 01 245 252 32618448 10.3290/j.ohpd.a43357 PMC11654493

[35] Pollick H The role of fluoride in the prevention of tooth decay Pediatr Clin North Am 2018 65 05 923 940 30213354 10.1016/j.pcl.2018.05.014

[36] Creeth J Bosma M L Govier K How much is a 'pea-sized amount'? A study of dentifrice dosing by parents in three countries Int Dent J 2013 63 02 25 30 24283281 10.1111/idj.12076 PMC9375012

[37] Liska D Kelley M Mah E 100% fruit juice and dental health: a systematic review of the literature Front Public Health 2019 7 190 31355175 10.3389/fpubh.2019.00190 PMC6640211

[38] Hooley M Skouteris H Boganin C Satur J Kilpatrick N Parental influence and the development of dental caries in children aged 0-6 years: a systematic review of the literature J Dent 2012 40 11 873 885 22842202 10.1016/j.jdent.2012.07.013

[39] Birant S Koruyucu M Ozcan H Investigating the level of knowledge of the community about oral and dental health Eur J Dent 2021 15 01 145 151 32932530 10.1055/s-0040-1716583 PMC7902119

